# Nasal Packing in Septal Surgery: A Narrative Review

**DOI:** 10.7759/cureus.36488

**Published:** 2023-03-21

**Authors:** Polina P Ivanova, Georgi Iliev

**Affiliations:** 1 Otolaryngology-Head and Neck Surgery, Saint Anna Hospital, Varna, BGR; 2 Otolaryngology-Head and Neck Surgery, University Hospital Saint Marina, Varna, BGR

**Keywords:** epistaxis, rhinoseptoplasty, intranasal splint, septoplasty, anterior nasal packing, nasal packing

## Abstract

Recently, alternatives to intranasal packing following septoplasty and rhinoseptoplasty have been widely used and promoted. Here, we aimed to systemically review and compare the different types of nasal packing used in these two surgeries. To assess patient comfort and surgical outcomes, we conducted a comprehensive search of multiple databases such as PubMed, MEDLINE, Web of Science, and Google Scholar to identify and evaluate relevant articles. A detailed and extensive search was performed with the help of the keywords "nasal packing," "septoplasty," "rhinoseptoplasty," "nasal splints," and "intranasal packing." Overall, our review findings indicate that alternative methods (e.g., trans-septal suturing and insertion of intranasal splints) are better options than intranasal packing.

## Introduction and background

Nasal obstruction is a common symptom observed in otorhinolaryngology. Although tolerance levels vary widely among patients, an obstructed nasal airway can generate anxiety and even claustrophobia, leading to a decreased quality of life. In most patients with impaired nasal breathing, the most common etiological factors leading to pronounced manifestation are septal deviation (76% of cases), nasal turbinate hypertrophy (72% of cases), and collapsed external nasal valve (67% of cases) [1], as well as other anatomical prerequisites such as septal spurs. Treatment typically involves septoplasty and surgical reduction of the nasal turbinates (i.e., conchotomy).

Bleeding from the nose, or epistaxis, can occur spontaneously due to systemic and local factors or postoperatively. Postoperative epistaxis can be controlled in a variety of ways, depending on the preferences of the surgeon, how easily the packing material can be placed and removed, cost-effectiveness, and patient comfort, particularly during removal. To date, however, an optimal choice for intranasal packing following rhinosurgery has not been identified [2].

## Review

Often patients undergoing rhinosurgery fear the postoperative period more than the surgery itself. [3] This is mainly due to the various types of intranasal packing and splints used after the operation. The purpose of intranasal packing and splints is to prevent the formation of synechiae and septal hematomas, to maintain the correct position of the newly shaped septum, to reduce dead space between the subperichondrial layers, and to control epistaxis. In cases where intranasal packing is necessary, patients report that the first five days of the postoperative period are the most unpleasant [4]. For the last two decades, transmucosal quilting sutures have been widely used as a reliable alternative to intranasal packing following septoplasty. Recent studies also recommend the use of intranasal splints instead of packing because splints offer better outcomes related to the frequency of bleeding, patient comfort, erosion prevention, nasal mucosa trauma, and synechiae.

Types of packing material

Intranasal packing is often applied in surgeries such as septoplasty, conchotomy, endoscopic sinus surgery, and rhinoseptoplasty. A wide variety of packing materials and shapes are used to perform hemostasis by compressing the local blood supply. In addition to halting bleeding, the packing creates a moist environment that favors mucosal healing without causing local irritation, injury, or immune responses to the foreign material.

Anterior nasal packing can be made of resorbable or non-resorbable material. A widely used non-resorbable packing material for the intranasal anterior consists of gauze strips covered in the lubricant or antibiotic ointment. Conventional resorbable anterior packing is made of polyurethane foam, such as Nasopore (Stryker, Kalamazoo, Michigan, U.S.), or hyaluronic acid, such as MeroGel (Medtronic, Minneapolis, Minnesota, U.S.) [5], which usually breaks down about a week after placement. The main disadvantage of resorbable materials is that they do not exert strong compression on the tissue and thus are not suitable for controlling severe hemorrhage or arterial bleeding. However, resorbable packing is better tolerated by patients [6], particularly as an anterior tamponade can be extremely uncomfortable and usually is kept in place for a minimum of 24 hours after surgery.

Some non-resorbable tamponades made from polyvinyl alcohol, for example Merocel (Medtronic, Minneapolis, Minnesota, U.S.) increase compression on blood vessels as they absorb fluid and increase in size, thus controlling arterial bleeding. Other packing models have a hollow tunnel made of polyvinyl alcohol, which serves as a canal to preserve nasal breathing. However, due to the continuous pressure exerted on all surfaces of the nasal mucosa and increased fluid absorption, the degree of mucosal trauma and bleeding may be greater with the use of non-resorbable absorbent packing, compared to resorbable options. One way to reduce mucosal trauma is to cover the non-resorbable packing with a latex finger from a surgical glove and suture the ends before placement. Another way is to use a purpose-designed material, such as the Netcell® Series 5000 (Network Medical, North Yorkshire, UK) tamponade, a polyvinyl alcohol sponge manufactured with a protective coating to prevent adhesion to tissue. Other non-resorbable tamponades, such as Arthrocare's Rapid Rhino, have built-in balloon zones that can be inflated with water or saline to provide additional compression to anticipated bleeding sites [7].

Bleeding from the back of the nasal cavity or nasopharynx requires a posterior tamponade, such as a classic Bellocq gauze posterior tamponade. The gauze bundle occludes the nasopharynx, and a bilateral anterior tamponade is placed. Another option for occlusion of the nasopharynx is the use of balloon tamponade, such as a Foley catheter 16F with a capacity of 30 ml per balloon. Specially designed balloon tamponades are available for simultaneous anterior and posterior placement, such as Summit's EpiStax™ (Summit Medical, Saint Paul, Minnesota, U.S.). This type of balloon packing has two outlets, one for the nasal cavity and one for the nasopharynx, each inflated separately. In cases of increased risk of postoperative bleeding or surgical complications, placement can be delayed up to 48 hours (Table 1).

**Table 1:** Packing Material

Packing Material	Type	Location	Products
Gauze	Non-resorbable	Anterior	
Splint	Non-resorbable	Posterior	
Balloon	Non-resorbable	Posterior and anterior	EpiStax™
Gauze	Non-resorbable	Posterior	Bellocq gauze
Polyvinyl alcohol sponge	Non-resorbable		Netcell® Series 5000 tamponade; Merocel
Hyaluronic acid	Resorbable		MeroGel
Polyurethane foam	Resorbable		Nasopore

Complications

For hemostasis of epistaxis, the usual duration of tamponade placement is two to four days, not to exceed five days [8]. Durations longer than five consecutive days can result in serious complications such as septal perforation, sinus infection, and necrosis of nasal mucosa [9]. The patient must breathe entirely through the mouth while the packing is in place, which can lead to dryness and sleep-disordered breathing. During this time, the risk of infection increases. Bacterial infections may result after 48 hours, as indicated by an unpleasant odor associated with tamponade removal [10]. Acute rhinosinusitis can develop if openings of the osteomeatal complex or sphenopalatine recess are obstructed by the tamponade itself or as a result of mucosal edema. Otitis media also can develop if a posterior tamponade obstructs the opening of the Eustachian tube [11]. Rarely, life-threatening complications can occur, such as bacteremia and toxic shock syndrome due to *Staphylococcus aureus*.

Most studies indicate that these types of complications occur most often with the use of non-resorbable intranasal tamponades [12]. Thus, patients undergoing rhinosurgery often receive prophylactic antibiotics prior to the procedure. However, this practice has been under debate recently, with no definitive answer yet from the literature. Most studies agree that standard prophylactic antibiotic administration for intranasal tamponade is clinically unjustified, in part because infection rates among patients who do not receive antibiotics are statistically insignificant [13,14].

The most common complications observed following intranasal packing are pain and discomfort during insertion and removal; breathing problems and hypoxemia; subsequent bleeding after removal; inability to achieve hemostasis; trauma or necrosis of the nasal mucosa, especially in the presence of septal deviation or bony spurs; necrosis of the columella and the alar zone, such as from pressure exerted by fixation of the posterior tamponade; local infection, such as rhinosinusitis, otitis media, and toxic shock syndrome; and displacement, migration, or aspiration of the tamponade [15-17].

Alternative methods and future directions

Intranasal tamponade placement has long been a part of post-rhinosurgical interventions, such as correcting a deviated nasal septum. Most physicians consider nasal packing to be a routine final stage of these surgeries, and studies have been assessing their efficacy since the 1970s [18,19]. Some have questioned the necessity of intranasal packing and instead sought viable alternatives. As Reiter et al. noted in their 1989 article “Alternatives to packing in septorhinoplasty,” intranasal tamponade is not a harmless procedure [19,20]. They assessed 75 rhinoplasty, septoplasty, and septorhinoplasty procedures and highlighted complications such as cardiovascular damage, prolonged bleeding, injury to the nasal mucosa, hypoxia, foreign body reaction, and infection. Reiter et al. further argued that intranasal packing could be avoided altogether with appropriate surgical methods, such as ensuring clean and precise mucosal incisions and avoiding crushing the tissues and tearing the mucosal ends for better suture fit [20]. They also highlighted the need for and importance of transmucosal (quilting, whipstitch) sutures for achieving appropriate tissue adaptation, closing dead space, and preventing blood accumulation and septal hematoma.

Nowadays, following septoplasty, each nasal cavity is routinely packed with an anterior tamponade, with different criteria regarding the material type and duration of placement [17,21]. For example, a Chinese study of 659 patients undergoing acoustic rhinometry found that nasal cavity volumes varied between 29,922 and 37,481 cm^3^, indicating that patient-specific tamponades were needed for adequate intranasal packing [22]. Salinger and Cohen were the first to use intranasal splints in septal surgery, which they cut from sheets of X-ray film [23]. Today, splints come in many sizes, shapes, configurations (with or without air tubes), and materials (plastic or silicone). They are placed on both sides of the nasal septum to prevent contact between the mucosa of the septum and mucosa of the lateral nasal wall and to facilitate postoperative maintenance of nasal septum stability [24].

The literature has not reached a consensus regarding the ideal intranasal tamponade or how long it should stay in place. Debates remain on the type of material, the placement technique, whether different tamponades are interchangeable, and whether they are even necessary at all. A 2015 study on 49 patients who underwent septoplasty found that those who received transmucosal sutures instead of intranasal tamponade had sufficient outcomes, with only three patients experiencing postoperative nasal synechiae [25]. A 2016 study of 92 patients compared the use of a tamponade (i.e., Merocel) with that of transmucosal sutures and observed no postoperative bleeding and statistically significantly lower levels of pain and headache in the group treated with transmucosal sutures; moreover, transmucosal sutures were more cost-effective than the tamponades [26].

A 2020 meta-analysis of 33 studies compared complications among septoplasty patients who received intranasal tamponade versus splints. It found that patients who received intranasal tamponade alone without splints had a higher incidence of complications, particularly nasal synechiae, compared to those who received splints. The authors also shared some other advantages of splints, noting that they support healing by moisturizing the mucosa, especially when mucosal integrity is disrupted; they mechanically protect the mucosa in the postoperative period and prevent further trauma; and they stabilize newly formed cartilage. However, splints were found to be more expensive than intranasal gauze tamponades or transmucosal sutures [27].

A 2021 retrospective study of 534 patients examined the risks and benefits of no tamponade after rhinoseptoplasty and, in cases of anterior tamponade (e.g., Merocel), the addition of intranasal splints. The tamponade was removed on the second postoperative day, and the splints on the seventh, during which patients were monitored for epistaxis, hematoma, septal perforation, impetigo, hyperalgesia, and dyspnea. The authors reported no significant difference in complications between the two patient groups: 4.4% of epistaxis cases were in the tamponade group, and 3% were in the non-tamponade group. Based on their findings, the authors recommended not placing intranasal tamponade as a safe alternative in rhinoseptoplasty procedures [28].

## Conclusions

Many variants of intranasal packing and splints have been developed over the years, all with advantages and disadvantages. Overall, the literature reviewed in this study indicates that septoplasty and rhinoseptoplasty can be safely performed with trans-septal stitching and insertion of silicone splints, instead of intranasal packing comprising gauze, polyvinyl alcohol, or resorbable material. However, we also acknowledge that the literature offered no established consensus regarding intranasal packing and splinting following septoplasty and rhinoseptoplasty.
